# How do primary care clinicians approach the management of frailty? A qualitative interview study

**DOI:** 10.1093/ageing/afae093

**Published:** 2024-05-05

**Authors:** Anna Seeley, Margaret Glogowska, Gail Hayward

**Affiliations:** Nuffield Department of Primary Care and Health Sciences, University of Oxford; Nuffield Department of Primary Care and Health Sciences, University of Oxford; Nuffield Department of Primary Care and Health Sciences, University of Oxford

**Keywords:** frailty, management, primary care, qualitative research, older people

## Abstract

**Background:**

Around 15% of adults aged over 65 live with moderate or severe frailty. Contractual requirements for management of frailty are minimal and neither incentivised nor reinforced. Previous research has shown frailty identification in primary care is *ad hoc* and opportunistic, but there has been little focus on the challenges of frailty management, particularly within the context of recent introduction of primary care networks and an expanding allied health professional workforce.

**Aim:**

Explore the views of primary care clinicians in England on the management of frailty.

**Design and setting:**

Semi-structured interviews were conducted with clinicians across England, including general practitioners (GPs), physician associates, nurse practitioners, paramedics and clinical pharmacists. Thematic analysis was facilitated through NVivo (Version 12).

**Results:**

A total of 31 clinicians participated. Frailty management was viewed as complex and outside of clinical guidelines with medication optimisation highlighted as a key example. Senior clinicians, particularly experienced GPs, were more comfortable with managing risk. Relational care was important in prioritising patient wishes and autonomy, for instance to remain at home despite deteriorations in health. In settings where more formalised multidisciplinary frailty services had been established this was viewed as successful by clinicians involved.

**Conclusion:**

Primary care clinicians perceive frailty as best managed through trusted relationships with patients, and with support from experienced clinicians. New multidisciplinary working in primary care could enhance frailty services, but must keep continuity in mind. There is a lack of evidence or guidance for specific interventions or management approaches.

## Key Points

- Frailty is best managed through trusted relationships with patients, and with support from experienced primary care clinicians.
- New multidisciplinary working in primary care could enhance frailty services, but must keep continuity in mind.
- There remains a large evidence gap to guide specific interventions and management approaches.

## Introduction

Frailty, as a ‘multidimensional syndrome characterised by decreased reserve and diminished resistance to stressors’ [1], is associated with increased healthcare utilisation [2], reduced quality of life [3] and life expectancy [4]. It affects 12% of those over 65 and at least 50% of those over 85 [5]. The majority of patients living with frailty will be managed by primary care clinicians.

The evidence base for frailty management in primary care is limited. Complex, multidimensional interventions such as the complex geriatric assessment (CGA) are the gold standard in secondary care, with high-quality evidence to suggest a reduction in length of stay, institutionalisation and mortality in hospitalised patients [6]. However these findings have not been replicated in trials based in primary care [7, 8]. Stronger evidence exists for strength training and nutritional supplementation [9], but it is unclear if these have been adopted into routine practice.

Historically there has been little formal policy or guidance on frailty management. The 2017/18 general practitioner (GP) General Medical Services contract introduced a falls assessment, medication review and cross-organisational record-sharing, for those living with severe frailty [10]. However, this was not accompanied by monitoring or financial incentivisation. Lengthier and more aspirational recommendations have been set out by NHS England but uptake is unclear [11]. Some local commissioning bodies provide additional funding for enhanced primary care services for frailty [12], suggesting there may be geographical variation in how care is delivered.

Finally, funding from NHS England via primary care networks (PCNs) has recruited 26,000 multidisciplinary professionals to general practice since 2019 [13]. Thus, there is potential scope for greater team-working and role-specialisation, which may benefit those living with frailty. However, alongside this there has been attrition of GP numbers [14] and a trend towards decreased continuity of care [15], amplified by the COVID-19 pandemic. Previous qualitative work, including our own research, has focused on how healthcare professionals identify frailty in primary care, rather than their experiences and beliefs regarding ongoing management [16–18]. We therefore sought to explore clinician experiences and perceptions of frailty management, within the complex and evolving landscape of UK primary care.

## Methods

### Design

Qualitative research methods were chosen given the lack of relevant literature and the suitability to explore both healthcare professionals’ experiences and perceptions of how they practice [19]. Semi-structured interviews were used so researchers could explore topics of interest in depth; together with issues participants themselves considered important [20].

### Recruitment

We recruited healthcare professionals who had been working within in primary care in England for at least 1 year, with regular contact with adults aged over 65. We recruited a mix of different professionals. This included GPs, advanced nurse practitioners (ANPs), physician associates (PAs), paramedic practitioners and clinical pharmacists. All of the professionals we recruited were able to autonomously consult with patients, diagnose new illness and manage long-term conditions. Some held prescribing or advance practitioner qualifications.

Clinicians were recruited through advertising on the Royal College of General Practitioners Research Surveillance Centre Network, social media and snowballing from other participants. Three participants were known in a professional capacity to the lead author (AES), prior to the study. Participants were offered a small honorarium (£80) for their time. Sampling was purposive, aiming to maximise variation in clinician experience, practice size, location and job role, including specialist interest in older people or frailty. Recruitment was ongoing until there was sufficient explanatory power for generated themes (i.e. our data had sufficient depth that our themes could blend and provide explanation for the experiences and perceptions expressed by our participants [21]) and there had been no amendments to topic guide or novel information for several interviews.

### Data collection

Interviews were conducted between April and September 2021 by lead author (AES), who has been trained in qualitative methods. Verbal and written consent were obtained prior to the interview. Interviews were conducted over telephone or Microsoft Teams and lasted between 30 and 70 min. Initial interviews were individually discussed with another researcher (MG). Data collection and analysis was concurrent, with regular discussion of findings between all authors. The topic guide (Supplementary File 1) was flexible and regularly reviewed and updated throughout data collection, as interviews suggested new themes or topics of interest.

### Data analysis

Interviews were transcribed verbatim by a university-approved transcribing service, and transcripts checked and anonymised. We used an inductive approach to reflexive thematic analysis [22] facilitated by Nvivo software (version 12) [23]. Interviews were coded by one researcher (AES). Analysis was guided by the constant comparative method [24], which involves reading and familiarisation with field notes and transcripts, with identification of initial themes, then open and systematic coding. The research team (AES, MG, GH) discussed an initial framework generated by initial coding of interviews. Critical discussion of identified categories and themes helped ensure credibility. Earlier interviews were re-coded in the light of new information or evolving themes.

### Patient and public involvement

Patient and public involvement (PPI) contributors were recruited based on their own experience of living, or caring for someone, with frailty. Four PPI contributors joined the team during the design of the study, and inputted during all stages of the project. PPI contributors explored how provisional results resonated with their own experiences and themes were reinterpreted in light of this discussion.

## Results

Of the 70 primary care clinicians who expressed interest in the study, 31, from 27 different practices, were chosen for interview, based on maximum diversity of job role and location. Table 1 displays participant characteristics. The number of ethnic minority patients registered at each practice varied from 1.5 to 82.9%, and average index of multiple deprivation varied from to the lowest to highest deciles of deprivation.

**Table 1:** Characteristics of participants

Job role	*N* = 31
GP partner	8
Salaried GP	4
ANP	4
Clinical pharmacist	7
Paramedic practitioner	2
PA	6
**Years of experience** ^1^	***N* = 31**
1–2	15
3–5	2
6–10	7
11–15	3
16–20	3
≥20	1
**Prescriber**	***N* = 30**
Yes	21
No	9
**Location**	***N* = 27**
Rural	1
Suburban	12
Urban	8
Mixed	6
**Practice size**	***N* = 27**
Small	2
Medium	5
Large	5
Very large	8
PCN	7
**Percentage of practice population over 65**	***N* = 27**
≤10%	5
11–15%	6
16–20%	7
21–25%	7
≥26%	2
**Specialist interest**	
Frailty specialist	8
Care home responsibilities	7
Home visits (not including GPs)	3

Main themes identified are displayed in Box 1 and discussed in further detail below.

**Box 1:** Main themes identified

Theme	Categories discussed
**Managing risk and complexity**	Role of primary care
	Clinician experience
**Relational care**	Advanced care planning
	Going above and beyond
	Threats to continuity
**Medication reviews**	Working within guidelines
	Trial and error
	Uncertainty about deprescribing
**Service organisation and coordination**	Formalised teams
	Social prescribers
	Availability of services
Use of specialists such as geriatricians	

### Theme 1: managing risk and complexity

The dominant discussion among primary care clinicians was how managing patients living with frailty was complex due to multiple competing conditions and priorities. A common dilemma encountered was when a patient presented with symptoms and signs suggestive of serious pathology, and a clinician had to weigh up the risks and benefits of further investigation. Clinicians discussed an awareness of the harms of over-diagnosis, but they perceived that this was often overlooked in clinical guidelines.

*‘I think that one of the most difficult things about frailty and older people is that you can’t just look it up in a book and follow NICE guidelines... it’s a really difficult decision’* (023, GP Partner, 6–10 years of experience).

Complexity also related to patients’ social circumstances. Often patients living with frailty were socially isolated, living in poverty or struggling to cope with activities of daily living. Participants described frustration at trying to communicate with family members who lived at a distance. One clinician described a sense of futility in trying to manage this complexity:

*‘Well, depends on your personality but my personality is being a bit of a fixer I’m afraid… if somebody’s lost their husband within the last few years, their family lives nowhere near, they’re getting less mobile,. You can’t fix it, so ... the tendency is to overlook it and focus on what you can fix to be honest’* (020—ANP, over 20 years of experience).

The accumulated experience of caring for patients living with frailty was an important factor in confidence in managing complexity. This was discussed across all roles, especially when clinicians reported a lack of frailty training or education.

*‘If you’ve got someone very frail ... who you think might have cancer... it might not be appropriate to refer them down the two week wait pathway. And something like that is completely foreign as a PA when you first qualify because you’re very much trained to diagnose and recognise cancer signs at a very basic level’* (031—Physician Associate, 6–10 years of experience).

There was some evidence that this complexity was viewed as beyond the remit of primary care, particularly in the context of acute illness. Diagnostic uncertainty coupled with potential for serious pathology was a motivating factor in referring patients for assessment to secondary care.

*‘So, if somebody calls and says, ‘Oh, my husband’s had a fall,’ depending on their age and what medication they’re on, I’d be like, ‘No, get them to call 999; he needs to go to hospital... it’s limited what a GP can do’* (019—Pharmacist, 1–2 years of experience).

A lack of confidence created anxiety in managing patients living with frailty, and fear of medico-legal repercussions if something went wrong. For this reason, a commonly expressed belief was that a senior clinician needed to be involved in the most difficult decisions. Senior GPs discussed the need to accept a level of personal risk and responsibility in doing what they felt was best for the patient.

*‘And I’ve seen that happen so many times where… older and more mature doctors who have that who think to themselves, ‘Well, in coroner’s court I’ll stand up to this.’ It’s very hard for someone who’s only qualified recently to think that they can do that.’* (025—GP Partner, 16–20 years of experience).

As a result, supervision and mentorship were important for inexperienced clinicians for some aspects of frailty management. This could create tension where not enough time was available for this activity, as it increased workload for GPs, while other clinicians felt unsupported.

*‘Sometimes you don’t get a response straight away, sometimes. . . they’ll [the doctor] be saying they haven’t got time to do it.* (017, Clinical Pharmacist, 1–2 years of experience).

*You lose faith in some of these additional staff; I do a little bit because I sometimes, to be honest, feel they generate more work than they solve problems.’* (004—GP Partner, 6–10 years of experience).

### Theme 2: relational care

Despite the complexity, clinicians gave many examples of how primary care managed frailty well. A holistic approach was often described i.e. placing a medical decision with the context of a patient’s life story and personal values. In general, this was less about level of frailty a patient was living with, but more about their individual needs. This was best exemplified in conversations about advanced care planning.

*‘So, a long time ago now, but seeing a ninety-three year old, who had COPD, who lived in a cottage without any electricity... and she just wanted to stay at home regardless of anything; never wanted to go to hospital. So, we had all put that down in paper ... she died very peacefully... Difficult when you’ve got no electricity’* (021—GP Partner, 11–15 years of experience).

Advanced care plans were mostly completed by GPs, except where other clinicians were given training, and knew patients well. Creating time to have these conversations could be challenging, but clinicians described real benefits for the patients, especially when done as early as possible. There was little reported resistance from patients, who often wanted to have these conversations with primary care clinicians.

*‘If I talk to a hundred people about advanced care planning there might only be one of them who says I don’t want to talk about this. The majority are very happy to talk about themselves and their life and their plans, and they feel privileged to do so. With somebody who they can trust.’* (010 Advanced Nurse Practitioner, 1–2 years of experience).

Participants observed a reduction in continuity of care, particularly driven by the COVID-19 pandemic, but also through the fragmentation of care within larger primary care teams. This was particularly so for GPs, who described losing touch with many of their older patients. The loss of relational care made it difficult to retain oversight and input on complex decisions. One GP partner suggested there needed to be new structures of working, with more regular communication between team members, to prevent this.

Where personal relationships did remain, participants gave several examples of going beyond their expected duties to provide high quality care.

*‘[I] probably went above and beyond in some respects because it was after my surgery on Friday evening… I spoke to the staff and said, ‘Look, you know, if we send her up to the hospital now for a chest x-ray, she’s going to sit…on a trolley on a ward probably all night.’ So, I went in on Saturday morning, reassessed her and arranged a local review with an x-ray at the General Hospital’* (023—GP Partner, 6–10 years of experience).

### Theme 3: medication reviews

Of the GMS contractual requirements, and NHS England policy recommendations, medication reviews were the intervention offered most consistently to patients living with frailty and conducted by GPs or pharmacists. Pharmacists performed lengthier structured medication reviews (SMRs), collecting additional information from patients to understand how well they were managing with their medications. These frequently picked up on wider problems necessitating GP review, or referral to other services.

*Oh yes, I ask her, ‘Can you walk around the house? Can you do the shopping? So, who’s helping you at home? So, who cooks; can you cook,’ for example. . . ‘Are you using a stick, a walking frame? Do you have a mobility scooter?’ ‘Do you manage to get up easily? Do you get dizzy in the morning? Do you need to hold on something while walking in the house?’* (014—Clinical Pharmacist, 1–2 years of experience).

Medication reviews helped identify potential harms from inappropriate prescribing in patients living with frailty. Deprescribing occurred most commonly when there was an adverse event, such as deterioration in renal function, clearly attributable to a medication. As such, participants felt that these changes were indicated irrespective of the patient’s age or frailty status.

Deprescribing also occurred across medication classes where this fitted with a tool or framework which supported medication reduction. Pharmacists who conducted SMRs were most familiar with these tools, such as the anticholinergic burden calculator. While clinicians found these tools helpful, deprescribing required greater discussion with the patient and often a ‘trial and error’ approach.

*I think it’s always difficult titrating down especially when someone’s finally feels like they’ve found their concoction that works for them. So, I always kind of try and provide reassurance, ‘Well if we try and take that a little bit. If your falls come back, it’s fine, you can go back up again, like don’t worry’.* (008—Clinical Pharmacist, 1–2 years of experience).

In contrast, opinion was divided as to when preventative medications, such as statins, could be stopped. Often pharmacists did not change statin prescriptions unless specific side effects were present. Many GPs described anxiety and uncertainty in deprescribing, even for those living with severe frailty, given the potential increased risk in cardiovascular events. Uncertainty was further compounded when medications were started or perpetuated in secondary care, and GPs felt it was particularly difficult to reverse these decisions. Consequentially deprescribing rarely happened until the very last stages of life, and GPs felt strongly that much clearer guidance was needed to change practice.

*‘We probably could do with more training around what could be stopped because…we feel a bit less confident in stopping things. We were told a statin doesn’t make a difference in the over eighties but people come out of hospitals on them, so you feel a bit confused - if I stopped them and then they get a stroke or a heart attack, could I be to blame?’* (006—GP Partner, 6–10 years of experience).

### Theme 4: service organisation

Six participants worked across four primary care organisations where there was a dedicated frailty service or team. These clinicians described similar organisational approaches which included holistic assessment to identify problems, proactive care and goal planning and onward referral where necessary. Overall, although these services were time- and resource-intensive, clinicians believed they resulted in high-quality care and clinical benefit. Services were mostly provided by non-medical clinicians who had developed frailty expertise, and worked with a high level of autonomy. Two of these organisations had developed services prior to 2019 but had harnessed PCN funding to secure ongoing employment of staff and creation of larger frailty teams. Integration of these teams within the primary care organisation meant they were easily accessible, flexible and responsive.

*‘A few weeks ago [the patient], who is now ninety-one, needed some support. He was getting his life in pickle… crawling up and down the stairs; his tablets were in an awful mess; he couldn’t get his stockings on and off; he wasn’t eating very well; he wasn’t self-caring very well and he couldn’t hear. So, I’m a bit of a whirling dervish. So, I addressed everything. So, he’s now got meals on wheels, he’s got an audiology appointment on Saturday, he has had OT and physio. He has moved his bed downstairs and he’s been to the Isle of Arran to spend a week with his daughter... he’s got his mojo back’* (022—ANP and lead of a PCN frailty service).

Although not as formalised, many clinicians working in other settings described multi-professional team-working. Social prescribers were available in more practices and seen as a valuable resource to help manage social complexities. In most cases this was to help signpost patients to other services e.g. befriending. In some instances, social prescribers had more regular contact with patients helping to reduce social isolation and the frequency of attendance at GP surgeries.

*‘And there’s certainly just patients… that just need a chat, a bit of reassurance every so often. So, she [social prescriber] will check on her every couple of weeks now, so it’s lessened our workload at the practice because it means that she’s calling less, less appointments and home visits’* (031—Physician Associate, 6–10 years of experience).

Communication between team members tended to be *ad hoc*, without formal coordination. For instance, most practices held multidisciplinary team meetings to discuss patients needing the most support; however clinicians reflected that patients living with frailty were rarely discussed, even in the context of acute deterioration. Meetings instead focused on cancer and palliative patients who were easy to identify from problem codes and required regular review in line with Quality Outcome Framework indicators.

‘*We have formal meetings once a month about the palliative patients, but not about elderly frail patients.’* (001—GP Partner, 16–20 years of clinical experience).

Referral to external community services targeting specific aspects of frailty, such as falls clinics, relied heavily on local availability. Few clinicians mentioned referring patients for nutritional support or structured exercise programmes. There appeared to be a lack of awareness as to what was available, or of the likely patient benefit. Participants expressed a desire for more information about existing pathways, as well as greater availability, especially for physical therapy programmes. Access to geriatricians was difficult as secondary care was busy, and there was a loss of a personal relationship between GPs and consultant colleagues. There was also a perception among some senior GPs that a geriatric review was of limited value:

*‘One of my patients had a review and it wasn’t that helpful actually because they [Geriatricians] sort of pose a lot of questions like, ‘Why are they on iron?’ or, ‘Why are they on this?’ Which I’d already thought of and there was a reason but had to look back and check, so it sort of generated work and didn’t change anything’* (004—GP Partner, 11–15 years of experience).

## Discussion

### Main findings

Managing patients living with frailty in primary care is complex, and commonly decisions fall outside of known evidence-base or clinical guidance. There was good evidence that primary care clinicians were well-placed to manage this complexity through both contextualising decisions to patient priorities, and using a multidisciplinary approach. Strong relationships with patients meant clinicians would go above usual responsibilities to try and align care with patient’s wishes, for example when avoiding admission. New roles in primary care, such as social prescribers, were used widely to tackle social vulnerabilities. Success appeared greater where practitioners had frailty expertise and used a holistic approach.

Clinicians identified several challenges. Decision-making often felt risky, with a fear of medico-legal repercussions and requiring oversight from senior GPs. Expansion of the primary care workforce coupled with current pressures had led to concerns that care had become fragmented with a loss of personal relationship. Finally, the gap in evidence as to how to best manage patients living with frailty in primary care led to uncertainty and inertia over specific clinical decisions, such as when to deprescribe, even among experienced clinicians.

### Strengths and limitations

To our knowledge, this is the first comprehensive study exploring how clinicians approach the management of frailty in UK primary care. We interviewed a diverse group of professionals reflecting current organisation of general practice [25].

Participation was limited to clinicians working in England, so results may not be transferable, although we sampled from a wide range of primary care organisations with different models of care. Participants may have had a greater knowledge in frailty management in responding to the advertisement, although only a minority declared a specialist interest or role. A total of 14 participants had less than 2 years’ experience in primary care, but this reflects the recent expansion of the workforce through creation of PCNs, and most had experiences working in other settings they could draw upon. We did not interview any non-clinical members of the primary care team including social prescribers. Given the evidence in our study of increasing involvement of social prescribers in the management of frailty this would be an interesting group to involve in future research. We did not conduct focus groups as we felt it was important to obtain greater depth through interviews, and we wanted to recruit diverse participants from different geographical areas. Given our findings and recent changes to how care is delivered, focus groups or case studies to understand more about the interactions between a multidisciplinary team could be of relevance in future studies. We also did not interview people living with frailty, as this was outside the scope of our research, but understanding their experiences further would be valuable in contextualising our findings.

### Comparison with existing literature

A handful of studies have explored approaches to frailty management in primary care or community staff. In Ireland, a recent study a mix of community professionals, including GPs, found that re-organisation and fragmentation of primary care had made it very challenging for older people living with frailty to navigate the healthcare system, something also evident in this study [26].

There is now considerable evidence, including our own research, that there is a lack of agreement within primary care, as to what frailty is, and how it should be recognised [16, 18, 27]. It is therefore unsurprising that the approach to management was similarly non-uniform and largely dependent on clinician’s role and experience. This finding was similarly replicated in a focus group study of community nurses in Scotland [28], and of a study of nine patients and their health professionals in the Netherlands [29]. There is a strong argument for addressing the identification of frailty to facilitate a more cohesive and structured approach. This may help improve access for people living with frailty to interventions that work. For instance, a recent systematic review found the most effective frailty interventions in primary care were structured exercise programmes and nutritional supplements [9], yet our research highlights that these interventions are rarely offered.

The uncertainty around optimising medications for patients living with frailty in our study mirrors previous study on deprescribing antihypertensives in older patients [30]. While GPs rely on ‘mindlines’, i.e. ‘*collectively reinforced, internalised tacit guidelines’* [31], in situations of ambiguity, pharmacists may not have this skill-mix without adequate training or experiential learning in practice [22]. Cohort studies have also highlighted variation in practice when deprescribing, particularly surrounding cardioprotective medications [32].

### Implications for policy and practice

Our research emphasises that primary care clinicians perceive the best way to manage frailty is through a therapeutic alliance with patients, with whom they have a trusted and personable relationship. This aligns with how older people want to experience healthcare [33], and arguably the essence of UK general practice [34]. However the ability of primary care to provide such relational care is increasingly eroded by declining GP numbers [14, 35], expansion of primary care teams, the introduction of digital triaging systems [36] and national policy focused on how quickly a patient is seen, rather than who they consult with [37]. Now is the time to rethink how continuity is provided. One solution may be to ring-fence those living with frailty as a group to prioritise for continuity, which in itself requires formalisation of the concept and identification of frailty in primary care [18]. But how continuity is achieved should also be directly addressed. A contemporary ontology of continuity in general practice divides this not only into therapeutic relationships, but illness episodes, distributed work and commitment to a community [38]. Addressing how primary care cares for those living with frailty using this framework may bring clarity for practitioners and policymakers alike, as to how this complex patient group can be managed in a complex and evolving health ecosystem.

Our study highlighted several success stories where holistic reviews and personalised care plans were delivered in GP practices or PCN teams. However, quantitative evidence for clinical outcomes, patient satisfaction or cost-effectiveness of these staff-intensive models is lacking. A Canadian feasibility study of a similar service provided through PCNs failed to show positive benefits on gait speed or patient related outcomes [39]. A recent feasibility study of modified CGAs delivieried within Scottish general practice suggested that this approach could reduce hospital admissions, but GP workload was also increased, over the first six months [40]. A better understanding is needed of what the key components of a `modified CGA' are, how these could be scaled up across PCNs and what additional resources are required for delivery. There is also a need to understand why older people living with frailty are not commonly referred to interventions which do work, such as strength training, and how to increase the acceptability of these interventions to clinicians and patients.

## Conclusions

Primary care clinicians perceive frailty as best managed through trusted relationships with patients, and with support from experienced clinicians. New multidisciplinary working in primary care could enhance frailty services, but must keep continuity in mind. There remains a large evidence gap to guide specific interventions or management approaches.

## Supplementary Material

aa-23-2091-File002_afae093

## Footnotes

1 Years of experience as an autonomous primary care practitioner. The shift in distribution to fewer years of experience reflects that most available CPclinical roles were not available prior to the introduction of Primary Care Networks in July 2019. Many of the CPs had many years of prior experience within other community settings from which they could draw on experiences of frailty.

## References

[1] Rodríguez-Mañas L , Féart C, Mann G et al. Searching for an operational definition of frailty: a Delphi method based consensus statement. The frailty operative definition-consensus conference project. J Gerontol A Biol Sci Med Sci 2013; 68: 62–7. 22511289 10.1093/gerona/gls119 PMC3598366

[2] Rockwood K , Howlett SE, Macknight C et al. Prevalence, attributes, and outcomes of fitness and frailty in community-dwelling older adults: report from the Canadian study of health and aging. The Journals of Gerontology: Series A 2004; 59: 1310–7. 10.1093/gerona/59.12.1310 15699531

[3] Crocker TF , Brown L, Clegg A et al. Quality of life is substantially worse for community-dwelling older people living with frailty: systematic review and meta-analysis. Qual Life Res 2019; 28: 2041–56. 30875008 10.1007/s11136-019-02149-1 PMC6620381

[4] Ensrud KE , Ewing SK, Taylor BC et al. Comparison of 2 frailty indexes for prediction of falls, disability, fractures, and death in older women. Arch Intern Med 2008; 168: 382–9. 18299493 10.1001/archinternmed.2007.113

[5] O’Caoimh R , Galluzzo L, Rodríguez-Laso Á et al. Prevalence of frailty at population level in European ADVANTAGE Joint Action Member States: a systematic review and meta-analysis. Ann Ist Super Sanita 2018; 54: 226–38. 30284550 10.4415/ANN_18_03_10

[6] Ellis G , Whitehead MA, Robinson D, O'Neill D, Langhorne P. Comprehensive geriatric assessment for older adults admitted to hospital: meta-analysis of randomised controlled trials. BMJ (Online) 2011; 343: d6553. 10.1136/bmj.d6553 PMC3203013 22034146

[7] Briggs R , McDonough A, Ellis G et al. Comprehensive geriatric assessment for community-dwelling, high-risk, frail, older people. Cochrane Database Syst Rev 2022; 2022: CD012705. 10.1002/14651858.CD012705.pub2 PMC9074104 35521829

[8] Garrard JW , Cox NJ, Dodds RM, Roberts HC, Sayer AA. Comprehensive geriatric assessment in primary care: a systematic review. Aging Clin Exp Res 2020; 32: 197–205. 30968287 10.1007/s40520-019-01183-w PMC7033083

[9] Travers J , Romero-Ortuno R, Bailey J, Cooney MT. Delaying and reversing frailty: a systematic review of primary care interventions. Br J Gen Pract 2019; 69: e61–9. 30510094 10.3399/bjgp18X700241 PMC6301364

[10] NHS England » GP Contract documentation 2017/18.

[11] NHS RightCare. *NHS Rightcare Frailty Toolkit.* June 2019.

[12] *Helping People Thrive Not Just Survive A Framework for Frailty in Dorset Supporting People in Dorset to Lead Healthier Lives*., 2017.

[13] Primary Care Network Workforce, 31 July 2023. *NHS Digital* 2023.

[14] The Health Foundation. *REAL Centre Projections: General Practice Workforce in England*., 2022.

[15] Tammes P , Morris RW, Murphy M, Salisbury C. Is continuity of primary care declining in England? Br J Gen Pract 2021; 71: E432–40. 33947666 10.3399/BJGP.2020.0935 PMC8103927

[16] Mulla E , Orton E, Kendrick D. Is proactive frailty identification a good idea? A qualitative interview study. Br J Gen Pract 2021; 71: e604–13. 33657008 10.3399/BJGP.2020.0178 PMC8252857

[17] Alharbi K , van Marwijk H, Reeves D, Blakeman T. Identification and management of frailty in English primary care: a qualitative study of national policy. BJGP Open 2020; 4: 1–12. 10.3399/bjgpopen20X101019 PMC7330193 32184213

[18] Seeley A , Glogowska M, Hayward G. ‘Frailty as an adjective rather than a diagnosis’—identification of frailty in primary care: a qualitative interview study. Age Ageing 2023; 52: 1–9. 10.1093/ageing/afad095 PMC10294554 37366329

[19] Pope C , Mays N. Qualitative Research in Health Care. 4th ed. Hoboken, New Jersey: Wiley Blackwell, 2020. 10.1002/9781119410867.

[20] Richie J , Lewis J, McNaughton Nicholls C et al. In: Jane R, Jane L, Carol MNN et al., eds. Qualitative Research Practice : A Guide for Social Science Students and Researchers, 2nd edition. Los Angeles: SAGE, 2014.

[21] Braun V , Clarke V. To saturate or not to saturate? Questioning data saturation as a useful concept for thematic analysis and sample-size rationales. Qual Res Sport Exerc Health 2019; 13: 201–16.

[22] Braun V , Clarke V. One size fits all? What counts as quality practice in (reflexive) thematic analysis? Qual Res Psychol 2021; 18: 328–52.

[23] Braun V , Clarke V. Using thematic analysis in psychology. Qual Res Psychol 2006; 3: 77–101.

[24] Silverman D . Doing Qualitative Research: A Practical Handbook. 5th edition, 2017.

[25] *General Practice and PCN Support The Primary Care Network Handbook 2021–22*.

[26] Kennedy F , Galvin R, Horgan NF. Managing Frailty in an Irish Primary Care Setting: A Qualitative Study of Perspectives of Healthcare Professionals and Frail Older Patients. 10.22540/JFSF-06-001. PMC8017349 33817445

[27] Ambagtsheer C , Archibald M, Lawless M, Mills D, Yu S, Bielby J. General practitioners’ perceptions, attitudes and experiences of frailty and frailty screening. Aust J Gen Pract 2019; 48: 426–33. 31256509 10.31128/AJGP-11-18-4757

[28] Papadopoulou C , Barrie J, Andrew M et al. Perceptions, practices and educational needs of community nurses to manage frailty. Br J Community Nurs 2021; 26: 136–42. 33719552 10.12968/bjcn.2021.26.3.136

[29] La Grouw Y , Bannink D, van Hout H. Care professionals manage the future, frail older persons the past. Explaining why frailty management in primary care doesn’t always work. Front Med (Lausanne) 2020; 7: 522013. 10.3389/fmed.2020.00489 PMC7485521 32984375

[30] Kuberska K , Scheibl F, Sinnott C et al. GPs’ mindlines on deprescribing antihypertensives in older patients with multimorbidity. Br J Gen Pract 2021; 71: e498–507. 34001537 10.3399/bjgp21X714305 PMC8249009

[31] Gabbay J , le May A. Evidence based guidelines or collectively constructed ‘mindlines?’ ethnographic study of knowledge management in primary care. BMJ 2004; 329: 1013. 15514347 10.1136/bmj.329.7473.1013 PMC524553

[32] Stewart D , Madden M, Davies P, Whittlesea C, McCambridge J. Structured medication reviews: origins, implementation, evidence, and prospects. Br J Gen Pract 2021; 71: 340–1. 34326073 10.3399/bjgp21X716465 PMC8312662

[33] Redding D , Gentry T, Shand J et al. I’m Still Me...A Narrative for Coordinated Support for Older People, 2014.

[34] Launer J , Greenhalgh PT. Narrative-Based Primary Care. CRC Press, 2017. 10.1201/9781315385549.

[35] Iacobucci G. England Lost 91 Fully Qualified Full Time GPs from Workforce Last Month, Data Show. 2022. 10.1136/bmj.o554. 35241428

[36] Ladds E , Khan M, Moore L, Kalin A, Greenhalgh T. The impact of remote care approaches on continuity in primary care: a mixed-studies systematic review. Br J Gen Pract 2023; 73: E374–83. 37105731 10.3399/BJGP.2022.0398 PMC10058181

[37] *The Future of General Practice Fourth Report of Session 2022–23 Report, Together with Formal Minutes Relating to the Report Health and Social Care Committee*., 2022.

[38] Ladds E , Greenhalgh T, Byng R, Rybczynska-Bunt S, Kalin A, Shaw S. A contemporary ontology of continuity in general practice: capturing its multiple essences in a digital age. Soc Sci Med 2023; 332: 116112. 37535988 10.1016/j.socscimed.2023.116112

[39] Abbasi M , Khera S, Dabravolskaj J, Chevalier B, Parker K. The seniors' community hub: an integrated model of care for the identification and management of frailty in primary care. Geriatrics 2021; 6: 1–14. 10.3390/geriatrics6010018 PMC8005937 33673051

[40] Jones HE , Anand A, Morrison I et al. Impact of MidMed, a general practitioner-led modified comprehensive geriatric assessment for patients with frailty. Age Ageing 2023; 52: 1–8. 10.1093/ageing/afad006 PMC10032632 36947740

