# Supplementary material for: How do primary care clinicians approach the management of frailty? A qualitative interview study
Source: Age Ageing. 2024 May 5;53(5):afae093. doi: 10.1093/ageing/afae093 (PMC11070720; doi:10.1093/ageing/afae093)
Supplement: aa-23-2091-File002_afae093 [file aa-23-2091-file002_afae093.docx]

**Identification and Management of Frailty in UK Primary Care: A Qualitative Interview Study**

Topic Guide for Semi-Structured Interviews

| Opening | Setting: practice population, demography, deprivation, workforce. Their role.  Can you tell me about a consultation with a patient you would describe as ‘frail?’ |
| --- | --- |
| Recognition | What makes that patient ‘frail’? Is that the same for every patient?  What does the term ‘frail’ mean to you?  Does anything about the patient’s social circumstances affect how you think about their frailty?  Does the method you use to consult affect the recognition of frailty e.g. telephone vs video vs face to face. How has this changed during COVID-19?  Who does the home visiting in your practice? What part do they play in your assessment of frailty?  How do you know who your frail patients are? Do you keep a list?  Is this something that you would formally code in the notes?   - What is this useful for (if at all?) - Why would you not code it? - Do you explain this to patients? If not why not?   Are there any organisational or financial factors which influence your recognition or coding of frailty?  Is it linked to QOF at all? |
| Stratification | Are you aware of the electronic frailty index?   - In what circumstances do you look at it / use it? - How useful do you find it? - If it was better would you use it anymore?   Do you recognise any different grades of frailty? Do you formally assess this with any tools?   - If not, what makes a person ‘severely frail’ rather than just frail?   Do you use the word frail with your patients? |
| Management | How do you manage frail patients?  Specific management points   - Do you routinely review medications of frail patients? Is a phamarcist involved? - How easy do you find it to deprescribe? - Would you routinely have conversations about DNACPR/advanced care planning with these patients? - Do you refer to falls clinic if falling? How useful if this service?   Do you have any specific services available to you for frail patients? How do these work? Are they useful?   - MDT meetings in practice - Link with DNs / Community Matron? - Link with secondary care?   Do you think frailty could be managed as a Long Term Condition like diabetes or asthma?  Does coding or identifying frailty benefit the patient’s care? |
| Future thoughts and closing | From our discussion is there anything that strikes you that could be changed or improved? How would this fit within your organisation or primary care network?  What resources would you need to make this change?  Will COVID-19 change our ability to assess or manage frailty? |
